# Cost-effectiveness analysis of tislelizumab plus chemotherapy versus placebo plus chemotherapy as first-line treatment for advanced gastric or gastroesophageal junction adenocarcinoma: perspectives from the United States and China

**DOI:** 10.3389/fphar.2024.1461571

**Published:** 2024-11-20

**Authors:** Wenwang Lang, Qi Ai, Wenwen Zhang, Qinling Jiang, Yulong He, Ming Ouyang

**Affiliations:** ^1^ Department of Pharmacy, Nanxishan Hospital of Guangxi Zhuang Autonomous Region, Guilin, China; ^2^ Department of Oncology, Nanxishan Hospital of Guangxi Zhuang Autonomous Region, Guilin, China; ^3^ Department Electrophysiology, Nanxishan Hospital of Guangxi Zhuang Autonomous Region, Guilin, China

**Keywords:** cost-effectiveness, unresectable gastric cancer, unresectable gastroesophageal junction cancer, tislelizumab, Markov model

## Abstract

**Purpose:**

The efficacy of tislelizumab plus chemotherapy in improving progression-free survival (PFS) and overall survival (OS) in unresectable gastric or gastroesophageal junction cancer (GC/GEJC) has recently been emphasized. This study compared the cost-effectiveness of tislelizumab plus chemotherapy versus placebo plus chemotherapy for the United States (US) and Chinese populations.

**Methods:**

Using data from the RATIONALE-305 phase 3 trial, a Markov model was developed to analyze quality-adjusted life years (QALYs), incremental cost-effectiveness ratios (ICERs), incremental net health benefits (INHBs), and incremental net monetary benefits (INMBs). The health state utilities and direct medical costs were obtained from the relevant literature and local cost databases. The model uncertainty was evaluated using sensitivity analyses.

**Findings:**

In the base-case analysis, the addition of tislelizumab to chemotherapy yielded an ICER of $37,768.48 per QALY in China, slightly below the willingness-to-pay (WTP) threshold of $38,042.49 per QALY, showing marginal cost-effectiveness with an INHB of 0.05 QALYs and an INMB of $1,852.49. Subgroup analyses revealed ICERs of $23,853.52 for patients with a PD-L1 TAP score ≥ 5% (TAP ≥ 5%). In the US, the ICER was $502,786.22 per QALY in the intent-to-treat (ITT) and $321,395.28 per QALY in the TAP ≥ 5% subgroup, exceeding the US WTP threshold of $150,000.00.

**Implications:**

In China, tislelizumab plus chemotherapy is a cost-effective first-line therapy for unresectable GC/GEJC in both ITT and TAP ≥ 5% subgroups. In the US, tislelizumab plus chemotherapy is not cost-effective.

## 1 Introduction

Gastric cancer (GC), including cancers at the gastroesophageal junction (GEJC), is the fourth most common cancer globally and a significant cause of cancer-related deaths. In 2020, it was responsible for approximately 1.1 million new diagnoses and more than 768,000 deaths (Sung et al., 2021). Standard care for unresectable, locally advanced, or metastatic GC or GEJC typically involves doublet or triplet therapy combinations of fluoropyrimidine and platinum-based chemotherapies. However, these therapies generally produce a median overall survival (OS) of only 1 year and are often associated with severe side effects (Van Cutsem et al., 2006; Roth et al., 2007; Cunningham et al., 2008; Ajani et al., 2010; Fuchs et al., 2019; Muro et al., 2019). Recent advances in immunotherapy, particularly those targeting the programmed death 1/programmed death-ligand (PD-1/PD-L1) axis, have shown promising results (Janjigian et al., 2021; Kang et al., 2022; Rha et al., 2023; Xu et al., 2023).

Tislelizumab, a PD-L1/PD-L2 signaling antagonist, has been noted for its ability to enhance cytokine production and reinvigorate T-cell function, which is crucial for the immune-mediated destruction of tumor cells (Zhang et al., 2018). The RATIONALE-305 clinical trial (Qiu et al., 2024) demonstrated that primary treatment with tislelizumab in combination with chemotherapy significantly improves OS compared to placebo with chemotherapy, particularly in patients with a PD-L1 tumor area proportion (TAP) ≥ 5% and among all participants. The combination of tislelizumab and chemotherapy was found to have a manageable and acceptable safety profile, positioning it as a viable initial treatment option for advanced GC/GEJC.

Compared with chemotherapy alone, nivolumab or pembrolizumab in combination with chemotherapy may not be a cost-effective option for treating advanced GC or GEJC in the US and China (Jiang et al., 2022; Shu et al., 2022; Cao et al., 2023; Lang et al., 2023; Marupuru et al., 2023; Zhang et al., 2023; Lang et al., 2024; Zheng et al., 2024). Tislelizumab is poised to redefine the treatment landscape for unresectable GC/GEJC in the US and China, potentially becoming the first globally approved anti-PD-1 antibody. Recently, tislelizumab has been marketed in countries and regions such as the US, UK, and EU. In China, tislelizumab costs approximately $400 per 21-day cycle, which is significantly lower than the costs of nivolumab and pembrolizumab. In the US, the price of tislelizumab is also approximately 15% lower than that of these therapies. Although tislelizumab has received approval in China and the US, its full impact on the global immunotherapy market for unresectable GC/GEJC remains unclear due to limited pricing information. This study evaluates the cost-effectiveness of combining tislelizumab with chemotherapy versus chemotherapy alone from US and Chinese healthcare payers’ perspectives to inform drug pricing strategies.

## 2 Patients and methods

### 2.1 Patient enrollment and intervention

The study followed the Consolidated Health Economic Evaluation Reporting Standards (Husereau et al., 2022). It enrolled patients aged 18 or older with histologically confirmed, locally advanced, unresectable, or metastatic gastric or gastro-oesophageal junction adenocarcinoma who had not received previous systemic therapy for their advanced condition. Participants who had undergone prior neoadjuvant or adjuvant treatment were eligible if they had experienced at least 6 months of disease-free progression. All patients were required to have an Eastern Cooperative Oncology Group (ECOG) performance status of 0 or 1 and at least one measurable or non-measurable lesion according to the Response Evaluation Criteria in Solid Tumors (RECIST) version 1.1, as assessed by the investigator. The enrollment was independent of the patient’s PD-L1 expression status.

Treatment involved a fixed dose of 200 mg tislelizumab or a corresponding placebo administered intravenously every 3 weeks. This was paired with the investigator’s choice of chemotherapy every 3 weeks: capecitabine at 1,000 mg/m^2^ twice daily on days 1–14 combined with oxaliplatin at 130 mg/m^2^ on day 1, or 5-fluorouracil at 800 mg/m^2^ on days 1–5 together with cisplatin at 80 mg/m^2^ on day 1, for a maximum of six cycles. Following this initial phase, patients continued with tislelizumab or placebo, with capecitabine maintenance allowed only for those initially treated with capecitabine and oxaliplatin until disease progression or unacceptable toxicity occurred. After 2 years of treatment, discontinuation of tislelizumab or placebo was considered if patients achieved a complete response, partial response, or stable disease, based on the investigator’s judgment of clinical benefit versus risk.

Patients in the study were eligible for second-line treatments, including ramucirumab plus paclitaxel, or they received the best supportive care. These treatment options were in line with guidelines from the National Comprehensive Cancer Network (NCCN) and the Chinese Society of Clinical Oncology (CSCO) for Gastric Cancer (2023) (Wang et al., 2024). The approach also reflected data from the RATIONALE-305 trial and standard clinical practices. Radiological tumor response assessments were performed using computed tomography (CT) or magnetic resonance imaging (MRI) following the RECIST version 1.1 guidelines. These assessments occurred approximately every 6 weeks for the first 48 weeks and then every 9 weeks.

The economic impact of adverse events (AEs) was evaluated using data from the RATIONALE-305 trial, focusing mainly on severe adverse events (SAEs) graded as 3 or 4. Only SAEs with a prevalence exceeding 3% were considered, including conditions such as anemia, reduced platelet and neutrophil counts, palmar-plantar erythrodysesthesia syndrome (hand-foot syndrome), and decreased appetite. To estimate the cost implications of these AEs, the incidence rate of each AE was multiplied by its respective unit cost of treatment, assuming that all AEs occurred within the initial treatment cycle. The incidences of these AEs are detailed in Table 1.

**TABLE 1:** Key clinical input data.

Parameters	Baseline value	Range		Distribution	Reference
		Minimum	Maximum		
Survival model for OS					
Tislelizumab plus chemotherapy	Shape = 1.5932 Scale = 15.1363			Loglogistic	Qiu et al. (2024)
Chemotherapy	Shape = 1.9040 Scale = 12.8902			Loglogistic	Qiu et al. (2024)
Survival model for PFS					
Tislelizumab plus chemotherapy	Shape = 1.5899 Scale = 7.6197			Loglogistic	Qiu et al. (2024)
Chemotherapy	Shape = 1.7759 Scale = 6.1872			Loglogistic	Qiu et al. (2024)
Survival model for OS (TAP ≥ 5)					
Tislelizumab plus chemotherapy	Shape = 1.5876 Scale = 16.8156			Loglogistic	Qiu et al. (2024)
Chemotherapy	Shape = 1.9450 Scale = 12.8650			Loglogistic	Qiu et al. (2024)
Survival model for PFS (TAP ≥ 5)					
Tislelizumab plus chemotherapy	Mu = 1.9084 Sigma = 1.1648 Q = −0.5822			Gengamma	Qiu et al. (2024)
Chemotherapy	Mu = 1.5805 Sigma = 0.9789 Q = −0.4974			Gengamma	Qiu et al. (2024)
Drug cost, $/per cycle					
Cost of Tislelizumab	355.78	284.62	426.94	Gamma	Local charge
Cost of Oxaliplatin	168.02	134.42	201.62	Gamma	Local charge
Cost of Capecitabine	44.53	35.62	53.44	Gamma	Local charge
Cost of 5-FU	128.22	102.58	153.86	Gamma	Local charge
Cost of Cisplatin	35.03	28.02	42.04	Gamma	Local charge
Cost of Paclitaxel	214.87	171.90	257.84	Gamma	Local charge
Cost of Ramucirumab	2,128.66	1702.93	2,554.39	Gamma	Local charge
Cost of Docetaxel	95.79	76.63	114.95	Gamma	Local charge
Cost of Nab-paclitaxel	553.45	442.76	664.14	Gamma	Local charge
Cost of Irinotecan	657.83	526.26	789.40	Gamma	Local charge
Cost of the laboratory test	106.61	85.29	127.93	Gamma	Zhang et al. (2019)
Enhanced CT	171.03	136.82	205.24	Gamma	Local charge
Cost of end-of-life	1,460.30	1,168.24	1752.36	Gamma	Wu et al. (2014)
Best supportive care	164.57	92.16	138.24	Gamma	Wu et al. (2014)
Cost of drug administration per unit	134.93	107.94	161.92	Gamma	Liu et al. (2023), Zhu et al. (2023)
Proportion of investigator’s choice of chemotherapy in Tislelizumab plus chemotherapy group					
Oxaliplatin and capecitabine	93.01%	74.41%	100.00%	Beta	Qiu et al. (2024)
Cisplatin and 5-fluouracil	6.99%	5.59%	8.39%	Beta	Qiu et al. (2024)
Proportion of investigator’s choice of chemotherapy in chemotherapy group					
Oxaliplatin and capecitabine	93.75%	75.00%	100.00%	Beta	Qiu et al. (2024)
Cisplatin and 5-fluouracil	6.25%	5.00%	7.50%	Beta	Qiu et al. (2024)
Proportion of receiving subsequent treatment					
Tislelizumab plus chemotherapy group	52.89%	42.31%	63.47%	Beta	Qiu et al. (2024)
Chemotherapy group	59.27%	47.42%	71.12%	Beta	Qiu et al. (2024)
Cost of AEs, $					
Anaemia	669.45	535.56	803.34	Gamma	Meng et al. (2022)
Decreased appetite	102.73	82.18	123.28	Gamma	Meng et al. (2022)
Decreased platelet count	1,054.22	843.38	1,265.06	Gamma	Meng et al. (2022)
Decreased neutrophil count	544.19	435.35	653.03	Gamma	Meng et al. (2022)
HFS	12.97	10.38	15.56	Gamma	Meng et al. (2022)
Utilities					
Utility of PFS	0.80	0.64	0.96	Beta	Shiroiwa et al. (2011)
Utility of PD	0.58	0.46	0.69	Beta	Shiroiwa et al. (2011)
Disutility estimates					
Anemia	0.07	0.06	0.084	Beta	Meng et al. (2022)
Decreased platelet count	0.11	0.09	0.132	Beta	Meng et al. (2022)
Decreased neutrophil count	0.20	0.16	0.240	Beta	Meng et al. (2022)
HFS	0.12	0.09	0.14	Beta	Meng et al. (2022)
Decreased appetite	0.08	0.062	0.09	Beta	Meng et al. (2022)
Risk for main AEs in Tislelizumab plus chemotherapy group					
Anemia	5.02%	4.02%	6.02%	Beta	Qiu et al. (2024)
Decreased platelet count	11.24%	8.99%	13.49%	Beta	Qiu et al. (2024)
Decreased neutrophil count	11.85%	9.48%	14.22%	Beta	Qiu et al. (2024)
HFS	3.01%	2.41%	3.61%	Beta	Qiu et al. (2024)
Decreased appetite	2.81%	2.25%	3.37%	Beta	Qiu et al. (2024)
Risk for main AEs in Chemotherapy group					
Anemia	7.49%	7.00%	10.50%	Beta	Qiu et al. (2024)
Decreased platelet count	11.54%	2.25%	3.37%	Beta	Qiu et al. (2024)
Decreased neutrophil count	11.54%	17.00%	25.50%	Beta	Qiu et al. (2024)
HFS	2.02%	15.00%	22.50%	Beta	Qiu et al. (2024)
Decreased appetite	3.24%	3.00%	4.50%	Beta	Qiu et al. (2024)
Discount rate	5.00%	4.00%	6.00%	Beta	
BMI/m^2^	1.72				
Weight/kg	65.00				
$1 = ¥7.05					

OS: overall survival, PFS: progression-free survival, PD: progression disease, TAP: PD-L1, tumor area proportion, AE: adverse event, BMI: body mass index, HFS: hand foot syndrome.

### 2.2 Model structure

**FIGURE 1:**
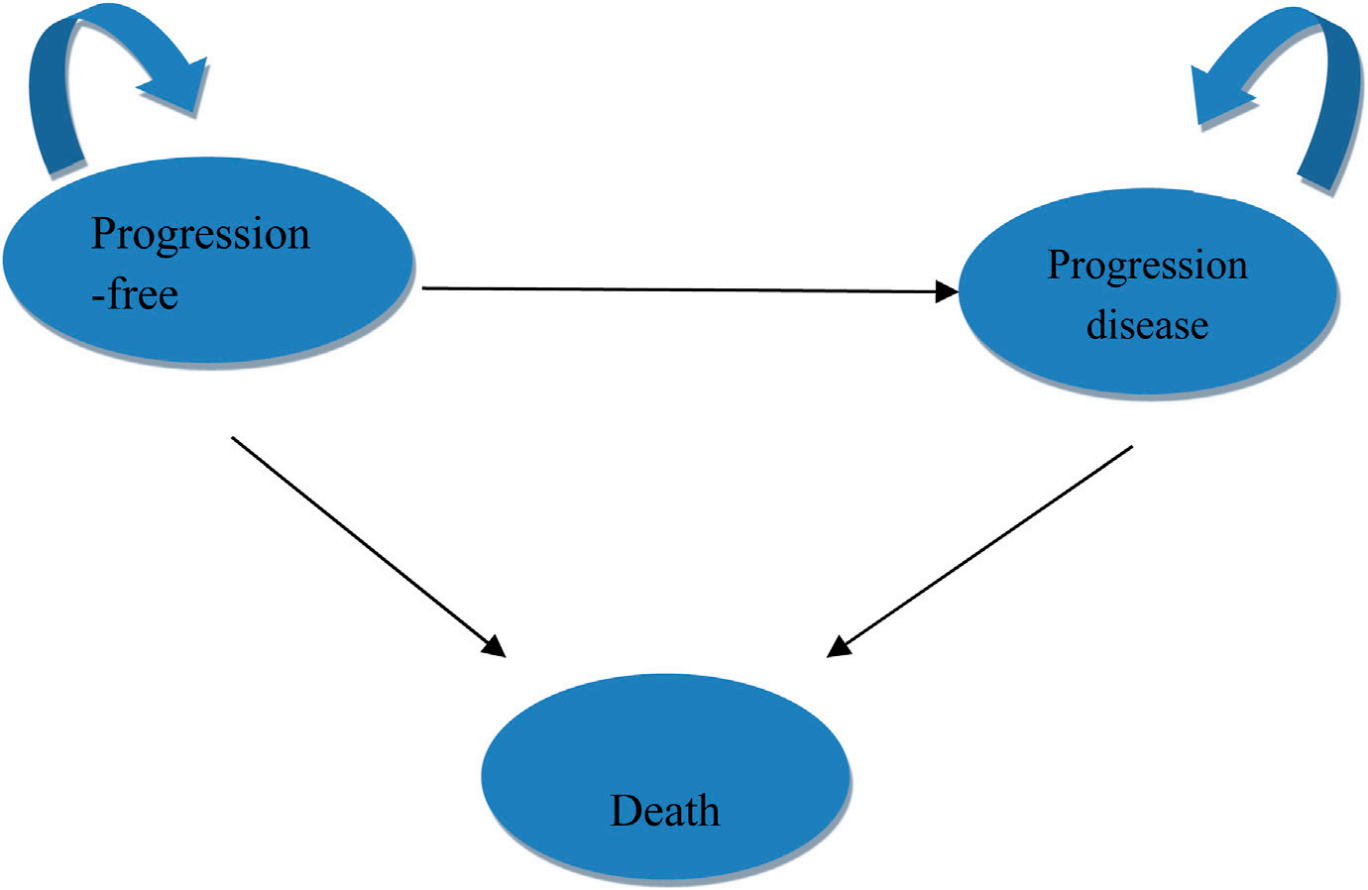
Markov model structure.

TreeAge Pro 2022 (Williamstown, MA, United States) and R software version 4.2.3 (Vienna, Austria) were used to develop and analyze the models. The core structure of the model was a three-state Markov model, including progression-free survival (PFS), progressive disease (PD), and death as distinct health states (Figure 1). Simulations were carried out over 10 years, capturing more than 99% of mortality events in both patient cohorts, with each simulation cycle lasting 3 weeks.

The analysis was conducted from the perspectives of healthcare payers in both the US and China. For the US, the analysis incorporated different funding sources, including public insurance, private insurance, and direct out-of-pocket expenses (Dieleman et al., 2020). In China, the perspective covered the entire healthcare system.

### 2.3 Outcomes

The primary outcomes were quality-adjusted life years (QALYs) and associated costs, both measured in US dollars. To account for time value, costs and utilities were discounted annually at 3% in the US and 5% in China (Su et al., 2021; Yue et al., 2021). The cost figures for China were adjusted to the 2023 values using the local consumer price index and converted to US dollars at a rate of $1 to ¥7.0467. The cost-effectiveness analysis used incremental cost-effectiveness ratios (ICERs), calculated with the formula: ICER = [Cost (tislelizumab plus chemotherapy) - Cost (placebo plus chemotherapy)]/[QALY (tislelizumab plus chemotherapy) - QALY (placebo plus chemotherapy)].

The willingness-to-pay (WTP) threshold was established at three times the *per capita* gross domestic product (GDP) of China for 2023, equating to $38,042.49, and at $150,000.00 for the US, according to World Health Organization (WHO) guidelines (Murray et al., 2000; Neumann et al., 2014). The analysis included assessments of incremental net health benefit (INHB) and incremental net monetary benefit (INMB), defined as follows: INHB (λ) = (μE1 - μE0) - (μC1 - μC0)/λ = ΔE - ΔC/λ and INMB (λ) = (μE1 - μE0) × λ - (μC1 - μC0) = ΔE × λ - ΔC. Here, μCi and μEi represent the cost and utility values associated with tislelizumab plus chemotherapy regimens (i = 1) and placebo plus chemotherapy (i = 0), respectively, while λ denotes the WTP threshold.

### 2.4 Clinical data inputs

The OS and PFS curves for the RATIONALE-305 trial were constructed using the algorithm developed by Guyot et al. (Murray et al., 2000). Time-to-event data points were extracted from the Kaplan-Meier curves for both OS and PFS using the GetData Graph Digitizer version 2.26 (www.getdata.graph.digitizer.com). These data points were used to model various parametric survival functions. The models tested were Gamma, Weibull, Exponential, WeibullPH (proportional hazards), Generalized Gamma, Gompertz, Log-normal, and Log-logistic distributions.

The selection of the optimal survival curves for both PFS and OS was based on a assessment of curve fit, incorporating both the Akaike Information Criterion (AIC) and Bayesian Information Criterion (BIC), along with a visual review of the fit results. The findings of these assessments are detailed in Tables 2, 3. Table 1 provides a summary of the derived shape (*g*) and scale (*l*) parameters for these survival models. The long-term survival trends are also shown in Supplementary Figures 1, 4.

**TABLE 2:** The Akaike information criteria (AIC) and bayesian information criteria (BIC).

Type of distribution	Tislelizumab plus chemotherapy (OS)		Placebo plus chemotherapy (OS)		Tislelizumab plus chemotherapy (PFS)		Placebo plus chemotherapy (PFS)	
	AIC	BIC	AIC	BIC	AIC	BIC	AIC	BIC
Exponential	3,073.373	3,077.590	3,190.154	3,194.360	2,623.539	2,627.755	2,605.722	2,609.928
Gamma	3,059.935	3,068.368	3,142.067	3,150.480	2,623.622	2,632.055	2,592.746	2,601.159
Generalized gamma	3,053.079	3,065.729	3,132.048	3,144.667	2,539.585	2,552.235	2,521.854	2,534.473
Gompertz	3,075.256	3,083.689	3,185.289	3,193.702	2,583.947	2,592.380	2,594.866	2,603.279
Weibull	3,064.916	3,073.349	3,154.027	3,162.440	2,625.195	2,633.628	2,603.969	2,612.382
WeibullPH'	3,064.916	3,073.349	3,154.027	3,162.440	2,625.195	2,633.628	2,603.969	2,612.382
Log-logistic	3,040.735	3,049.168	3,119.800	3,128.213	2,527.995	2,536.428	2,519.063	2,527.476
Lognormal	3,065.127	3,073.560	3,136.923	3,145.33	2,541.685	2,550.118	2,523.609	2,532.022

OS: overall survival; PFS: progression-free survival.

**TABLE 3:** The akaike information criteria (AIC) and bayesian information criteria (BIC) (TAP ≥ 5% subgroup).

Type of distribution	Tislelizumab plus chemotherapy (OS)		Placebo plus chemotherapy (OS)		Tislelizumab plus chemotherapy (PFS)		Placebo plus chemotherapy (PFS)	
	AIC	BIC	AIC	BIC	AIC	BIC	AIC	BIC
Exponential	1,632.570	1,636.183	1,723.683	1,727.289	1,419.243	1,422.856	1,421.272	1,424.877
Gamma	1,625.130	1,632.356	1,694.863	1,702.075	1,420.872	1,428.099	1,415.824	1,423.036
Generalized gamma	1,616.114	1,626.953	1,682.704	1,693.522	1,365.866	1,376.705	1,363.996	1,374.814
Gompertz	1,634.568	1,641.794	1723.084	1730.296	1,396.870	1,404.097	1,411.926	1,419.138
Weibull	1,628.670	1,635.897	1703.720	1710.932	1,420.631	1,427.858	1,422.002	1,429.214
WeibullPH'	1,628.670	1,635.897	1703.720	1710.932	1,420.631	1,427.858	1,422.002	1,429.214
Log-logistic	1,611.795	1,619.022	1,677.305	1,684.516	1,371.926	1,379.152	1,366.263	1,373.475
Lognormal	1,614.637	1,621.863	1,680.774	1,687.985	1,372.544	1,379.770	1,368.658	1,375.869

OS: overall survival; PFS: progression-free survival; TAP: PD-L1, tumor area proportion.

### 2.5 Cost inputs

We focused exclusively on direct medical costs, which included a variety of expenses related to the treatment and management of gastric and gastro-oesophageal junction adenocarcinoma. These costs included expenditures for medications, laboratory tests, routine imaging such as chest and abdomen enhanced CT, prophylactic treatments associated with each intravenous administration, best supportive care, end-of-life care, costs related to drug administration, subsequent treatments, and the management of severe (grade 3 and 4) AEs. The pricing information for the medications was obtained from public databases and local pricing schedules. At the same time, other cost data was derived from a review of previously published studies and other relevant literature.

The drug doses used in the RATIONALE-305 trial protocol served as the basis for calculating the cost of each treatment cycle. These costs were determined by applying the prescribed dosage schedules to current local pricing structures (Table 1). The costs associated with managing each AE were calculated by multiplying the incidence of each AE by the cost required to treat it (Table 1). Tables 1, 4 summarize the essential clinical input data that informed the cost analysis (Wu et al., 2014; Zhang et al., 2019; Liu et al., 2021; Zhang et al., 2021; Meng et al., 2022; Liu et al., 2023; Shao et al., 2023; Zhang et al., 2023; Zhu et al., 2023; Centers for Medicare and Medicaid Services, 2024).

**TABLE 4:** Key clinical input data (US).

Parameters	Baseline value	Range		Distribution	Reference
		Minimum	Maximum		
Drug cost, $/per cycle					
Cost of Tislelizumab	8,640.00	6,912.00	10,368.00	Gamma	Centers for Medicare and Medicaid Services (2024)
Cost of Oxaliplatin	39.31	31.45	47.17	Gamma	Centers for Medicare and Medicaid Services (2024)
Cost of Capecitabine	109.90	87.92	131.88	Gamma	Centers for Medicare and Medicaid Services (2024)
Cost of 5-FU	50.17	40.14	60.20	Gamma	Centers for Medicare and Medicaid Services (2024)
Cost of Cisplatin	45.79	36.63	54.95	Gamma	Centers for Medicare and Medicaid Services (2024)
Cost of Paclitaxel	40.45	32.36	48.54	Gamma	Centers for Medicare and Medicaid Services (2024)
Cost of Ramucirumab	6,268.77	5,015.02	7,522.52	Gamma	Centers for Medicare and Medicaid Services (2024)
Cost of Docetaxel	182.91	146.33	219.49	Gamma	Centers for Medicare and Medicaid Services (2024)
Cost of Nab-paclitaxel	7,487.84	5,990.27	8,985.41	Gamma	Centers for Medicare and Medicaid Services (2024)
Cost of Irinotecan	128.92	103.14	154.70	Gamma	Centers for Medicare and Medicaid Services (2024)
Cost of the laboratory test	111.65	89.32	133.98	Gamma	Centers for Medicare and Medicaid Services (2024)
Enhanced CT	424.35	339.48	509.22	Gamma	Centers for Medicare and Medicaid Services (2024)
Cost of end-of-life	21,603.00	17,282.40	25,923.60	Gamma	Shao et al. (2023)
Best supportive care	3,049.00	2,439.20	3,658.80	Gamma	Shao et al. (2023)
Cost of drug administration first hour	142.55	114.04	171.06	Gamma	Liu et al. (2021)
Administration intravenous, additional hour	30.68	24.54	36.82	Gamma	Liu et al. (2021)
Cost of AEs, $					
Anemia	7,941.00	6,352.80	9,529.20	Gamma	Shao et al. (2023)
Decreased platelet count	13,105.00	10,484.00	15,726.00	Gamma	Shao et al. (2023)
Decreased neutrophil count	13,105.00	10,484.00	15,726.00	Gamma	Shao et al. (2023)
HFS	8,382.19	6,705.75	10,058.63	Gamma	Zhang et al. (2021)
Decreased appetite	12,874.84	10,299.87	15,449.81	Gamma	Zhang et al. (2021)
Discount rate	3.00%	0.02	0.04	Beta	
BMI/m^2^	2.10				
Weight/kg	75.00				

AE: adverse event, BMI: body mass index, HFS: hand foot syndrome.

### 2.6 Quality-of-life inputs

The health utility scores were evaluated on a scale ranging from 0 (death) to 1 (perfect health). Due to the lack of directly available European Quality of Life-5 Dimensions-5 Level (EQ-5D-5L) data from the RATIONALE-305 trial, alternative and robust sources were utilized to estimate quality-of-life measurements.

The utility value for patients in the PFS phase was determined to be 0.797. Using the Japanese scoring algorithm, this estimate was derived from the EQ-5D responses collected during the TOGA trial (Shiroiwa et al., 2011). In the case of PD, the utility value was set at 0.577 (Shiroiwa et al., 2011), based on evaluations from the National Institute for Health and Clinical Excellence.

AEs were assumed to negatively impact health utility (referred to disutility). In the model, the disutility associated with AEs was accounted for only during the first treatment cycle. These utility values and pertinent quality-of-life inputs are documented in Table 1 (Meng et al., 2022).

### 2.7 Scenario analysis

Due to significant uncertainties regarding model assumptions and parameter sources, scenario analyses were integral to this research. To assess the impact of price fluctuations on cost-effectiveness, the price of tislelizumab in the model varied from $0 to $500 per 100 mg in China and from $0 to $2,500 in the United States This range was explored to determine potential cost-effectiveness under WTP thresholds set at $38,042.49 and $150,000.00. Given the significant influence of subsequent treatments on the results, scenario analyses were conducted by varying the proportions and types of subsequent treatments. To evaluate the impact of these subsequent treatments on cost-effectiveness, patients were assumed to receive either additional treatment or the best supportive care. The second-line chemotherapy regimens, which are Category 1 recommendations in the guidelines, were assessed, including paclitaxel, docetaxel, nab-paclitaxel, and irinotecan.

### 2.8 Subgroup analyses

The cost-effectiveness of tislelizumab combined with chemotherapy versus placebo combined with chemotherapy as the primary treatment for unresectable GC/GEJC was evaluated using subgroup analyses focused on patients with a TAP ≥ 5%. These analyses used the same methodologies as base case analysis, including one-way (OWSA) and probabilistic sensitivity analyses (PSA). Due to the lack of specific data from the RATIONALE-305 trial regarding subsequent treatment choices, drug selections, and AE occurrences for patients with TAP ≥ 5%, these factors were assumed to mirror those observed in the broader trial population.

### 2.9 Sensitivity analysis

Model uncertainty was assessed using two methods of sensitivity analysis: OWSA and PSA. In OWSA, the parameter values were derived from the literature and adjusted by ±20% relative to their baseline values. A comprehensive approach was used for PSA, involving the simultaneous adjustment of all parameters in 10,000 Monte Carlo simulations. This method facilitated the evaluation of cost-effectiveness probabilities for various interventions against different WTP thresholds for an QALY. Utility parameters were modeled using beta distributions, and gamma distributions represented cost parameters. The results were visually presented through scatter plots and cost-acceptability curves to convey the key outcomes effectively.

## 3 Results

### 3.1 Base case analysis

Over 10 years, base-case outcomes demonstrated that the group receiving tislelizumab plus chemotherapy gained an additional 1.00 QALYs at an increased cost of $28,297.65. In contrast, the chemotherapy-only group accrued 0.78 QALYs with costs of $20,263.25. Comparative analysis revealed a mean incremental gain of 0.21 QALYs at an additional cost of $8,034.40 for the tislelizumab group. This led to an ICER of $37,768.48 per QALY for combination therapy compared to chemotherapy alone (Table 5). When assessed against China’s WTP threshold of $38,042.49 per QALY, combination therapy was more cost-effective than chemotherapy alone, with an INHB of 0.002 QALYs and an INMB of $58.29 (Table 5). In contrast, in the US, the ICER for tislelizumab plus chemotherapy was $502,786.22 per QALY, which exceeded the US WTP threshold of $150,000.00 per QALY. The analysis also indicated an INHB of −0.53 QALYs and an INMB of $-80,200.47 relative to chemotherapy alone at the same WTP threshold (Table 5).

**TABLE 5:** The base case analysis.

Treatment	Cost	QALY	Incremental cost	Incremental QALY	INHB	INMB	ICER
Tislelizumab plus chemotherapy (China)	28,297.65	1.00	8,034.40	0.21	0.002	58.29	37,768.48
Chemotherapy (China)	20,263.25	0.78					
Tislelizumab plus chemotherapy (TAP ≥ 5%) (China)	27,746.58	1.16	8,594.51	0.36	0.13	5,112.34	23,853.52
Chemotherapy (TAP ≥ 5%) (China)	19,152.07	0.80					
Tislelizumab plus chemotherapy (US)	188,096.92	1.03	114,300.64	0.23	−0.53	−80,200.47	502,786.22
Chemotherapy (US)	73,796.28	0.80					
Tislelizumab plus chemotherapy (TAP ≥ 5%) (US)	193,023.84	1.20	123,696.77	0.38	−0.44	−65,965.63	321,395.28
Chemotherapy (TAP ≥ 5%) (US)	69,327.07	0.82					

QALY: Quality-adjusted life year, ICER: Incremental cost-effectiveness ratio, INMB: the incremental net monetary benefits, INHB: the incremental net health benefits, TAP: PD-L1, tumor area proportion.

### 3.2 Price simulation

**FIGURE 2:**
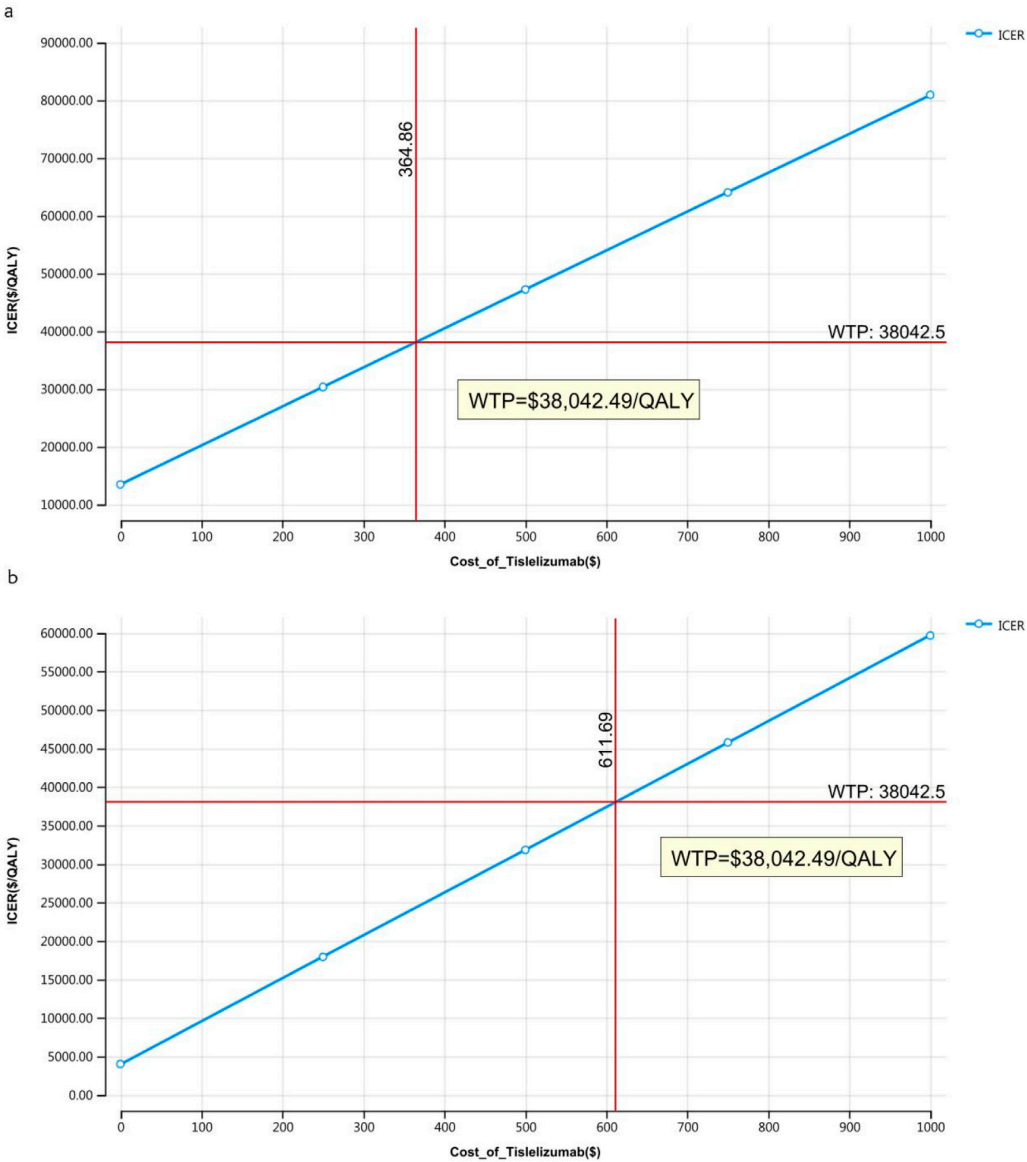
Price simulation of China **(A)** Patients with ITT, **(B)** Patients with TAP ≥ 5%.

**FIGURE 3:**
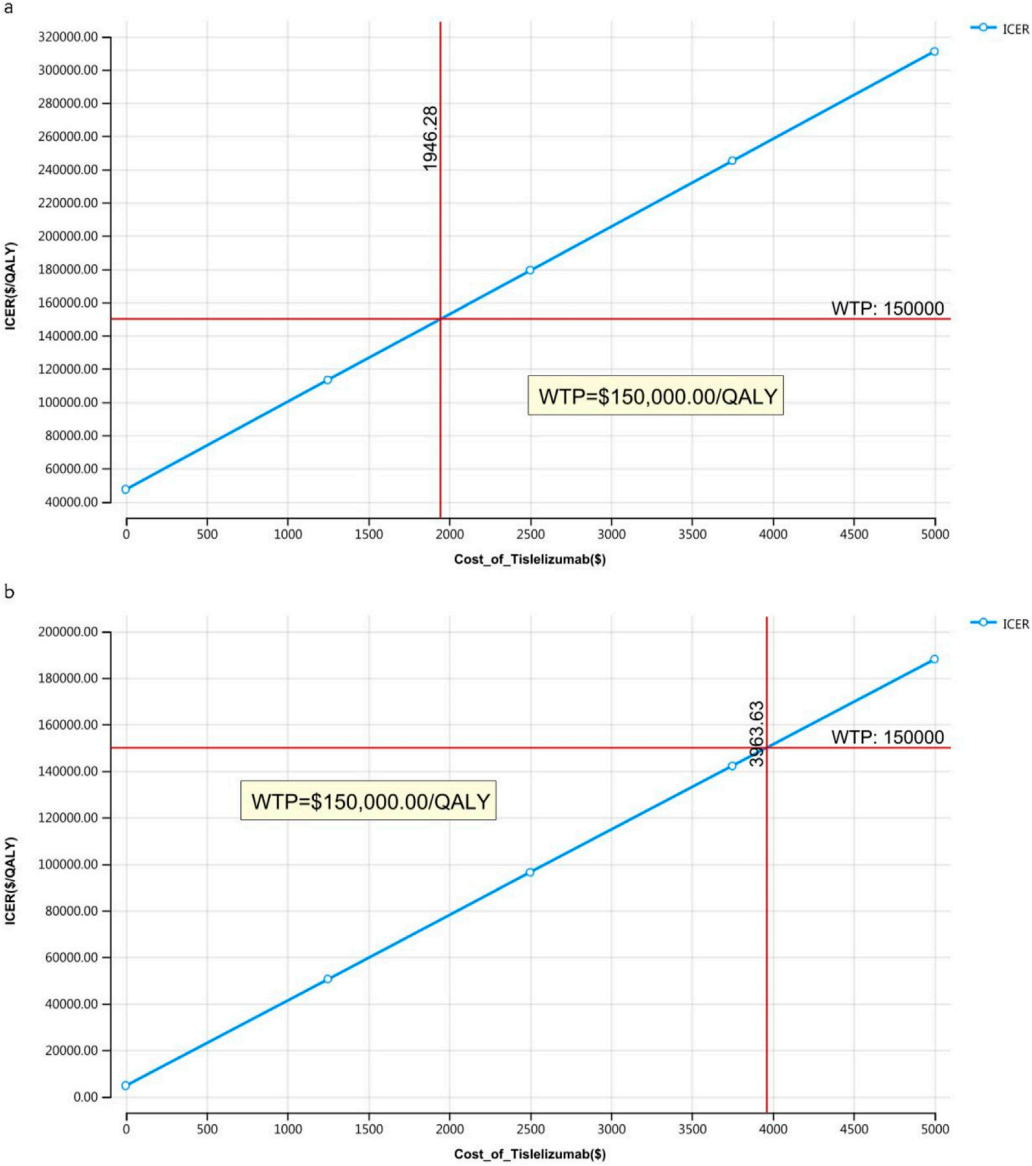
Price simulation of the United States **(A)** Patients with ITT, **(B)** Patients with TAP ≥ 5%.

The outcomes of the price simulation, depicted in Figures 2, 3, show that as the price of tislelizumab varied from $0 to $500.00 per 100 mg in China and from $0 to $2,500.00 per 100 mg in the US, the ICER increased accordingly. Tislelizumab remained cost-effective when priced below $182.43 per 100 mg for the intent-to-treat (ITT) group and $305.85 per 100 mg for the subgroup with TAP ≥ 5% in China, according to a WTP threshold of $38,042.49. In the US, the corresponding cost-effectiveness thresholds were $973.14 per 100 mg for the ITT group and $1,981.82 per 100 mg for the subgroup with TAP ≥ 5%, based on a WTP threshold of $150,000.00. The results of the scenario analyses, which varied the proportions and types of subsequent treatments, are presented in Table 6.

**TABLE 6:** The base case analysis.

Types of receiving subsequent treatment	ICER(China)	PSA (China)	ICER(US)	PSA (US)
Ramucirumab plus paclitaxel	53,655.01	2.23%	527,134.42	0
Ramucirumab plus paclitaxel (TAP ≥ 5%)	26,580.24	88.91%	325,541.95	0.17%
Best supportive care	32,172.27	79.24%	493,362.97	0
Best supportive care (TAP ≥ 5%)	27,274.97	87.62%	326,171.44	0.11%
Paclitaxel	32,620.25	76.59%	485,883.39	0
Paclitaxel (TAP ≥ 5%)	27,001.07	88.24%	329,962.44	0.09%
Docetaxel	32,332.25	77.45%	486,267.51	0
Docetaxel (TAP ≥ 5%)	27,177.15	87.82%	329,767.75	0.09%
Nab-paclitaxel	33,439.12	71.82%	505,964.20	0
Nab-paclitaxel (TAP ≥ 5%)	26,500.43	88.83%	319,784.53	0.39%
Irinotecan	33,691.57	70.52%	486,121.93	0
Irinotecan (TAP ≥ 5%)	26,346.09	88.61%	329,841.54	0.10%

ICER: Incremental cost-effectiveness ratio, PSA: probabilistic sensitivity analyses.

### 3.3 Subgroup analysis

Subgroup analyses indicated that the ICER for tislelizumab plus chemotherapy compared to placebo plus chemotherapy was $23,853.52 per QALY gained for patients with TAP ≥ 5% in China, and $321,395.28 per QALY gained in the United States These ICER values was below the WTP thresholds of $38,042.49 per QALY in China and above $150,000.00 per QALY in the US (Table 5). The INHB of tislelizumab plus chemotherapy for patients with TAP ≥ 5% was 0.13 QALYs in China and −0.44 QALYs in the United States The INMB at the WTP thresholds of $38,042.49 per QALY and $150,000.00 per QALY was $5,112.34 and $-65,965.63 in China and the US, respectively, compared to placebo plus chemotherapy (Table 5).

### 3.4 Sensitivity analysis

**FIGURE 4:**
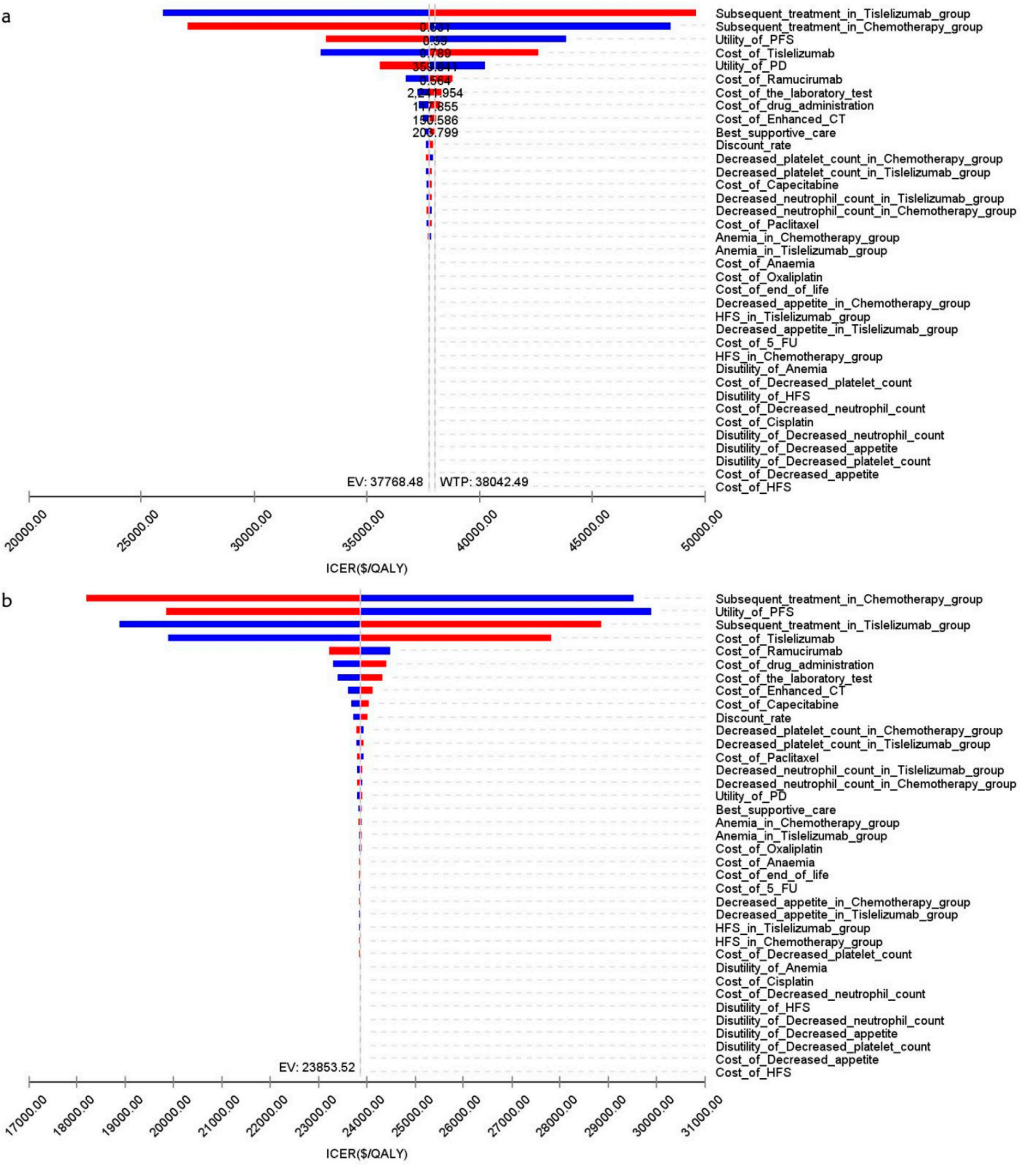
The tornado diagram of one-way sensitivity analysis in China **(A)** Patients with ITT, **(B)** Patients with TAP ≥ 5%.

Figure 4 presents a tornado diagram from OWSA for the Chinese population, emphasizing the factors that most significantly influence the base-case outcomes. Key variables included the number of patients who received subsequent treatment in the overall cohort, the TAP ≥ 5% subgroup, and the PFS utility values, which significantly impacted the base-case results.

**FIGURE 5:**
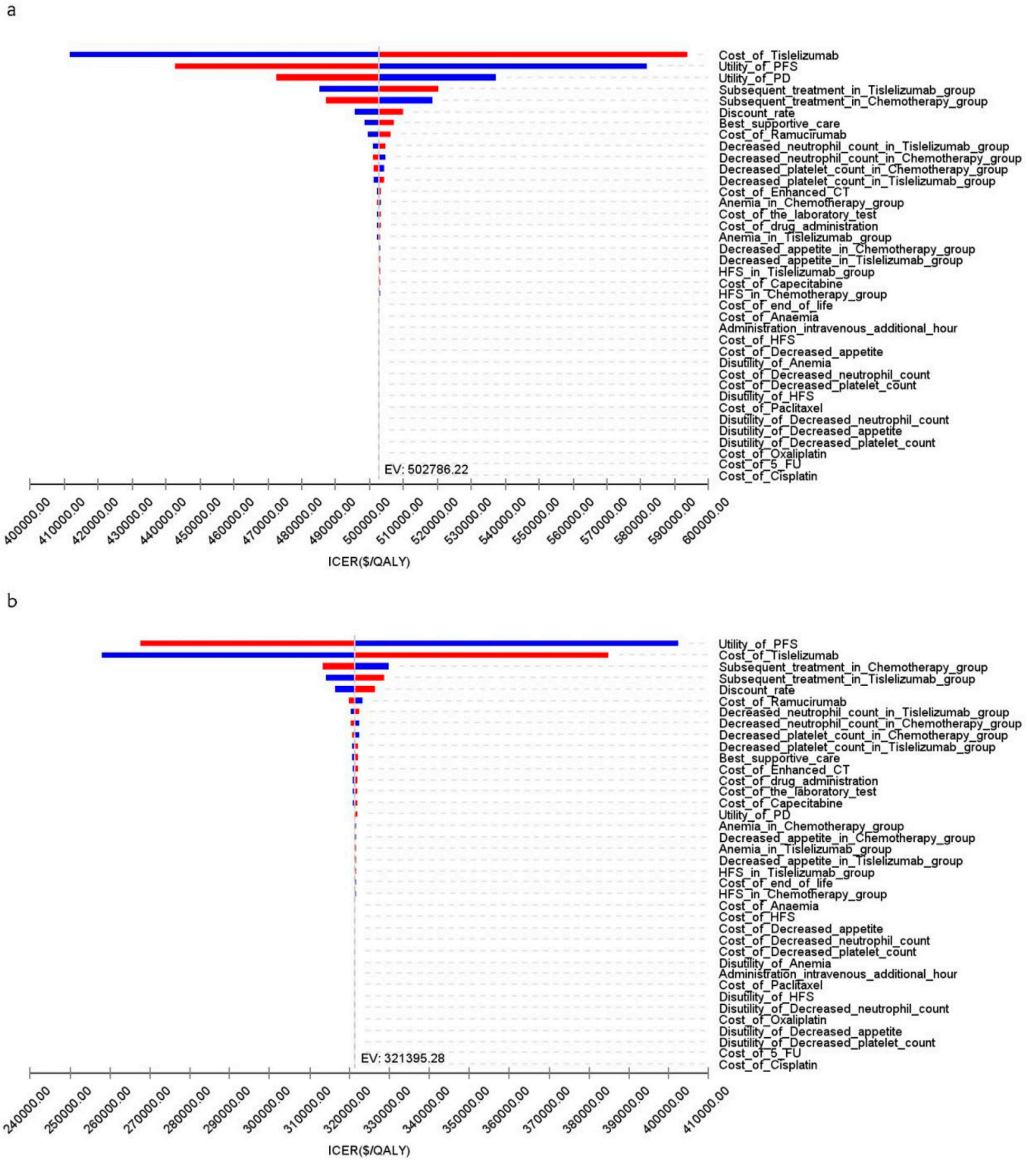
The tornado diagram of one-way sensitivity analysis in the US **(A)** Patients with ITT, **(B)** Patients with TAP ≥ 5%.

Figure 5 illustrates that, for patients in the US, the primary factors influencing the ICER across all patients were the costs of tislelizumab, PFS utility, and PD utility. For the TAP ≥ 5% subgroup, the ICER was primarily influenced by the costs of tislelizumab, PFS utility, and the number of patients receiving subsequent treatment in the chemotherapy group. However, due to the significant differences in health outcomes between the two treatment strategies, variations in these parameter values did not materially affect the overall study results.

**FIGURE 6:**
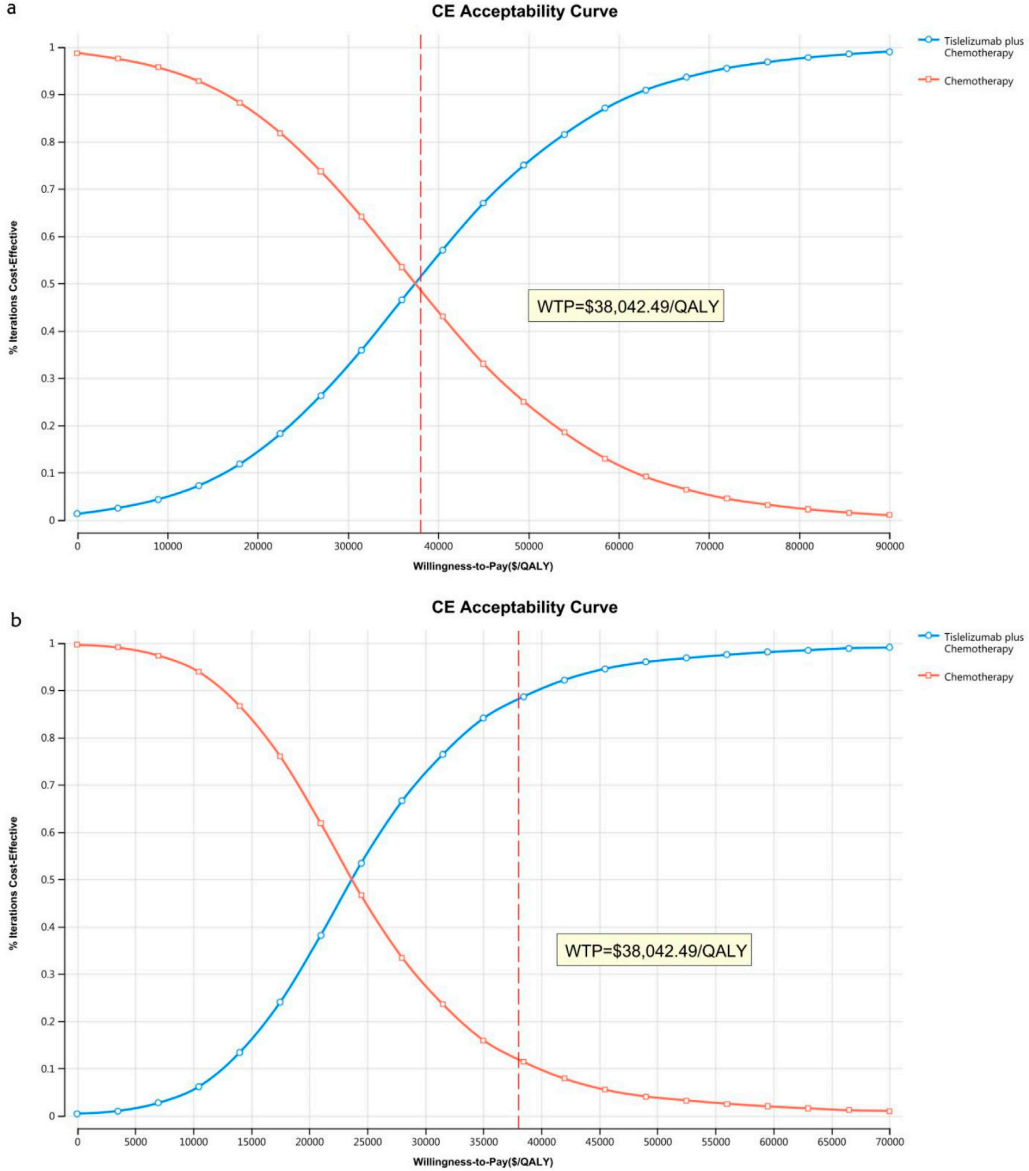
The cost-effectiveness acceptability curve in China **(A)** Patients with ITT, **(B)** Patients with TAP ≥ 5%.

**FIGURE 7:**
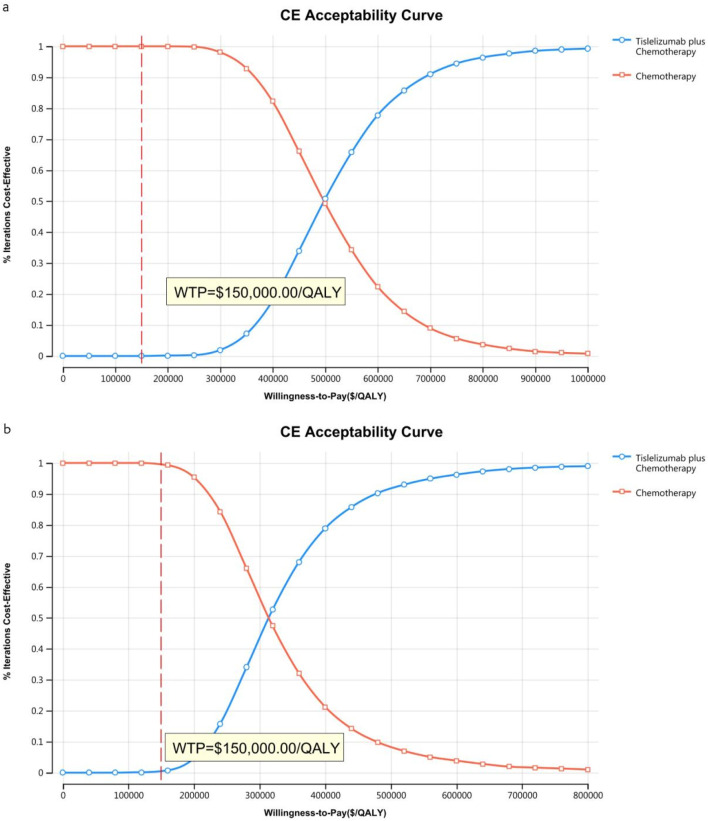
The cost-effectiveness acceptability curve in the US **(A)** Patients with ITT, **(B)** Patients with TAP ≥ 5%.

**FIGURE 8:**
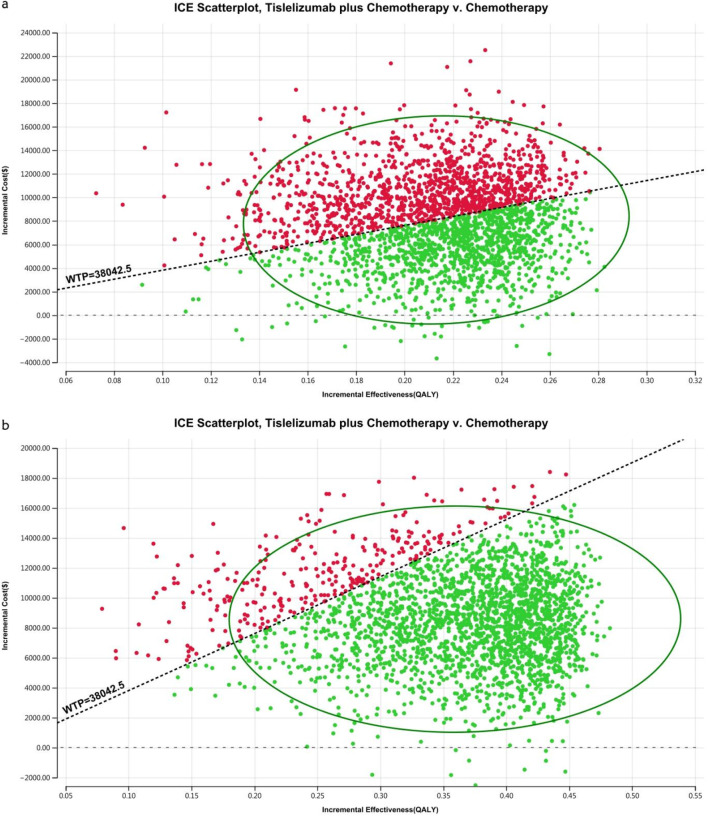
The cost-effectiveness probabilistic scatter plot in China **(A)** Patients with ITT, **(B)** Patients with TAP ≥ 5%.

**FIGURE 9:**
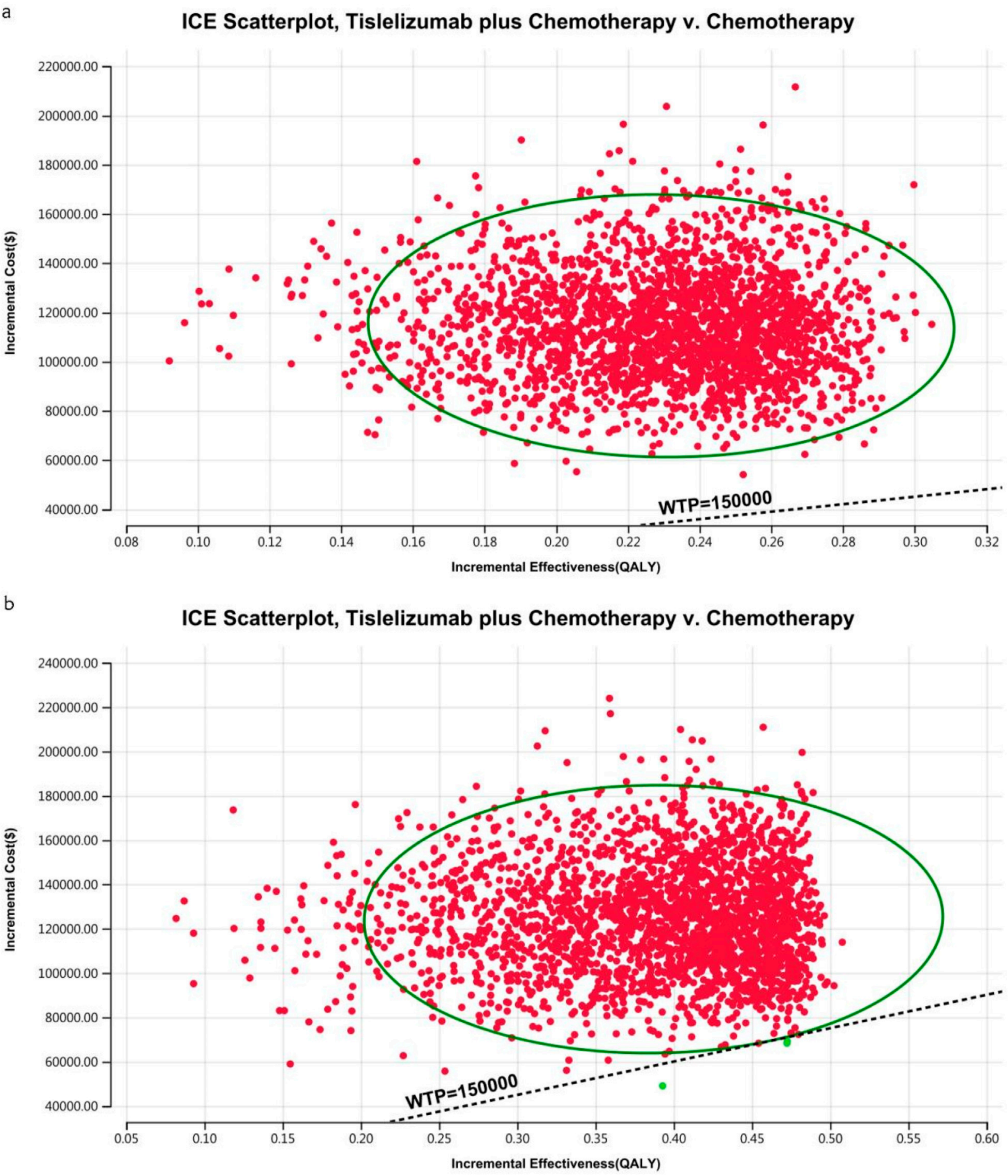
The cost-effectiveness probabilistic scatter plot in the US **(A)** Patients with ITT, **(B)** Patients with TAP ≥ 5%.

The acceptability curves and probabilistic scatter plots that map out the cost-effectiveness landscape are shown in Figures 6–9. Cost-effectiveness probabilities for tislelizumab plus chemotherapy were indicated by the PSA to be 51.44% for the entire cohort and 88.49% for the TAP ≥ 5% subgroup in China. In the US, these probabilities were determined to be 0% for the entire cohort and 0.32% for the TAP ≥ 5% subgroup, relative to a WTP threshold of three times China’s GDP *per capita* ($38,042.49) and $150,000.00 in the US, respectively.

## 4 Discussion

The RATIONALE-305 trial demonstrated that adding tislelizumab to chemotherapy improved PFS, OS, and other critical efficacy outcomes in patients with unresectable GC/GEJC. The ambiguity challenges healthcare providers and patients in assessing its cost-effectiveness, which requires comprehensive economic evaluation.

From the perspective of the Chinese healthcare system, our economic analysis based on the RATIONALE-305 trial indicates that tislelizumab combined with chemotherapy produces an ICER of $37,768.48 per QALY for ITT patients and $23,853.52 per QALY for the TAP ≥ 5% subgroup. These figures fall below China’s WTP threshold of $38,042.49 per QALY. Nivolumab or pembrolizumab combined with chemotherapy as a first-line treatment for individuals with advanced GC or GEJC has generally not demonstrated superior cost-effectiveness compared to chemotherapy alone in China, as supported by previous studies (Jiang et al., 2022; Shu et al., 2022; Lang et al., 2023; Zhang et al., 2023; Lang et al., 2024; Zheng et al., 2024). Our findings highlight that the first Chinese-made immune checkpoint inhibitor, tislelizumab, is a cost-effective option for the treatment of unresectable GC/GEJC.

However, in China, the National Healthcare Security Administration (NHSA) has engaged in multiple negotiation rounds with pharmaceutical companies, primarily focusing on re-evaluating anticancer drug pricing structures. These negotiations have significantly reduced the prices of various anticancer drugs, enhancing the cost-effectiveness of tislelizumab plus chemotherapy from the perspective of Chinese healthcare. Our price simulations suggest that tislelizumab remains economically favorable when priced below $182.43 per 100 mg for the ITT group and $305.85 per 100 mg for the subgroup with TAP ≥ 5%. This is not a very high price, and the policy and clinical practice need to consider this issue to benefit more patients carefully.

No immune checkpoint inhibitors combined with chemotherapy for advanced GC or GEJC have demonstrated superior cost-effectiveness compared to chemotherapy alone in the US(Cao et al., 2023; Marupuru et al., 2023; Zhang et al., 2023). Our findings are consistent with these results. Since tislelizumab has only recently been launched in the US, price simulations were conducted, showing that tislelizumab remains economically favorable when priced below $973.14 per 100 mg for the ITT group and $1,981.82 per 100 mg for the TAP ≥ 5% subgroup, based on a WTP threshold of $150,000.00 in the United States Lowering the price of immune checkpoint inhibitors is essential to expanding patient access and ensuring more patients benefit from these treatments. With the significant reduction in the cost of tislelizumab, our study’s results are promising and indicate potential benefits for a broader patient population. These findings underscore the importance of drug pricing negotiations in improving the accessibility and cost-effectiveness of advanced cancer therapies.

The Kaplan-Meier curves for OS and PFS significantly influence outcomes. Following an additional 17-month follow-up, updated data for patients with TAP of ≥ 5% indicated significant improvements in the tislelizumab plus chemotherapy arm versus the placebo plus chemotherapy arm. For OS, median survival was 16.4 months (95% CI 13.6–19.1) compared to 12.8 months (95% CI 12.0–14.5) for the placebo group, with a stratified hazard ratio of 0.71 (95% CI 0.58–0.86). For PFS, the hazard ratio was 0.68 (95% CI 0.56–0.83).

PD-L1 expression is systematically evaluated by a central laboratory utilizing the TAP score. This score quantifies the proportion of the tumor area (comprising both tumor cells and any associated desmoplastic stroma) that displays PD-L1 staining on tumor cell membranes at any intensity, as well as on tumor-associated immune cells. The evaluation uses the investigational version of the Ventana PD-L1 (SP263) assay from Roche Diagnostics. The prevalence of patients with TAP ≥ 5% in the RATIONALE-305 trial was similar to that observed in other studies for advanced GC/GEJC (Janjigian et al., 2021; Xu et al., 2023). Encouragingly, our study demonstrated an acceptable concordance rate between TAP ≥ 5% and combined positive scores (CPS) of ≥5 in exploratory analyses. Similar OS results with tislelizumab plus chemotherapy were observed in subgroups defined by a TAP score or a combined positive score. The OS benefit of tislelizumab plus chemotherapy was more pronounced in patients with TAP ≥ 5% compared to those with lower scores, suggesting that higher PD-L1 expression could enrich survival benefits. Subgroup analysis focusing on patients with TAP ≥ 5% revealed an ICER of $23,853.52 in China and $321,395.28 per QALY gained in the US, lower than that of the ITT population and consistent with the clinical findings of the RATIONALE-305 trial. These results support the targeted use of tislelizumab in patients with increased PD-L1 expression to optimize cost-effectiveness and therapeutic outcomes in advanced GC/GEJC.

The sensitivity analysis of the RATIONALE-305 trial indicates that the ranking of influencing factors in China and the United States is inconsistent. This discrepancy may be due to the substantial cost differences between the two countries and the varying discount rates applied, which lead to significant differences in QALY and increased costs. Additionally, the impact of second-line treatment is a significant determinant, ranking within the top three factors affecting the outcomes of experimental studies. In the trial, subsequent anticancer therapies were administered to 53% of patients receiving tislelizumab plus chemotherapy, 50% receiving additional chemotherapy, and 30% undergoing targeted therapy, the most common treatment. In comparison, 59% of the patients in the placebo plus chemotherapy group received subsequent treatments, including 57% who underwent additional chemotherapy and 32% who received targeted therapy, again the most common options. Following the guidelines of the NCCN and the Chinese Society of Clinical Oncology for gastric cancer (2023), the combination of ramucirumab plus paclitaxel was selected as the latest treatment regimen. The scenario analysis, which varied the types of subsequent treatments, suggested that as treatment prices decreased, the ICER increased in the ITT group. However, the relationship was more complex in the TAP ≥ 5% subgroup, possibly due to the use of indirect data in this subgroup.

This study has several limitations. First, the simulation model is based on clinical trial data, which introduces inherent uncertainties due to the reliance on such data, a common challenge for models of this type. Despite this, the model’s alignment with survival data was confirmed by testing eight different distributions, as supported by the sensitivity analysis. Second, the absence of specific data from the RATIONALE-305 trial on the subsequent treatment plans, drug selection, and AE occurrences for patients with TAP ≥ 5% required assumptions that these parameters align with observations from the general patient population. According to the RATIONALE-305 trial, the TAP ≥ 5% subgroup demonstrated a better remission rate, required fewer follow-up treatments, and experienced fewer AEs. As a result, the ICER for this subgroup was likely overestimated. Third, OWSA highlighted the ICER’s sensitivity to utility values. However, PFS and PD utilities had to be obtained from the existing literature due to the lack of EQ-5D-5L scale data from the RATIONALE-305 trial, potentially introducing bias due to inconsistency between study participants. Despite these challenges, the cost-effectiveness analysis based on the RATIONALE-305 trial offers valuable information, enhancing the flexibility of practical clinical therapies and supporting informed treatment decision-making.

## 5 Conclusion

Our study findings highlight the superior cost-effectiveness of tislelizumab plus chemotherapy compared to placebo plus chemotherapy when used as a first-line therapy for the entire patient cohort and for patients with TAP ≥ 5% with unresectable GC/GEJC within the Chinese healthcare system. But this combination therapy does not demonstrate cost-effectiveness for patients in the United States

## Data Availability

The original contributions presented in the study are included in the article/Supplementary Material, further inquiries can be directed to the corresponding author.
