# Supplementary material for: Cost-effectiveness analysis of tislelizumab plus chemotherapy versus placebo plus chemotherapy as first-line treatment for advanced gastric or gastroesophageal junction adenocarcinoma: perspectives from the United States and China
Source: Front Pharmacol. 2024 Nov 20;15:1461571. doi: 10.3389/fphar.2024.1461571 (PMC11614636; doi:10.3389/fphar.2024.1461571)
Supplement: Supplementary file 3 [file Image1.pdf]

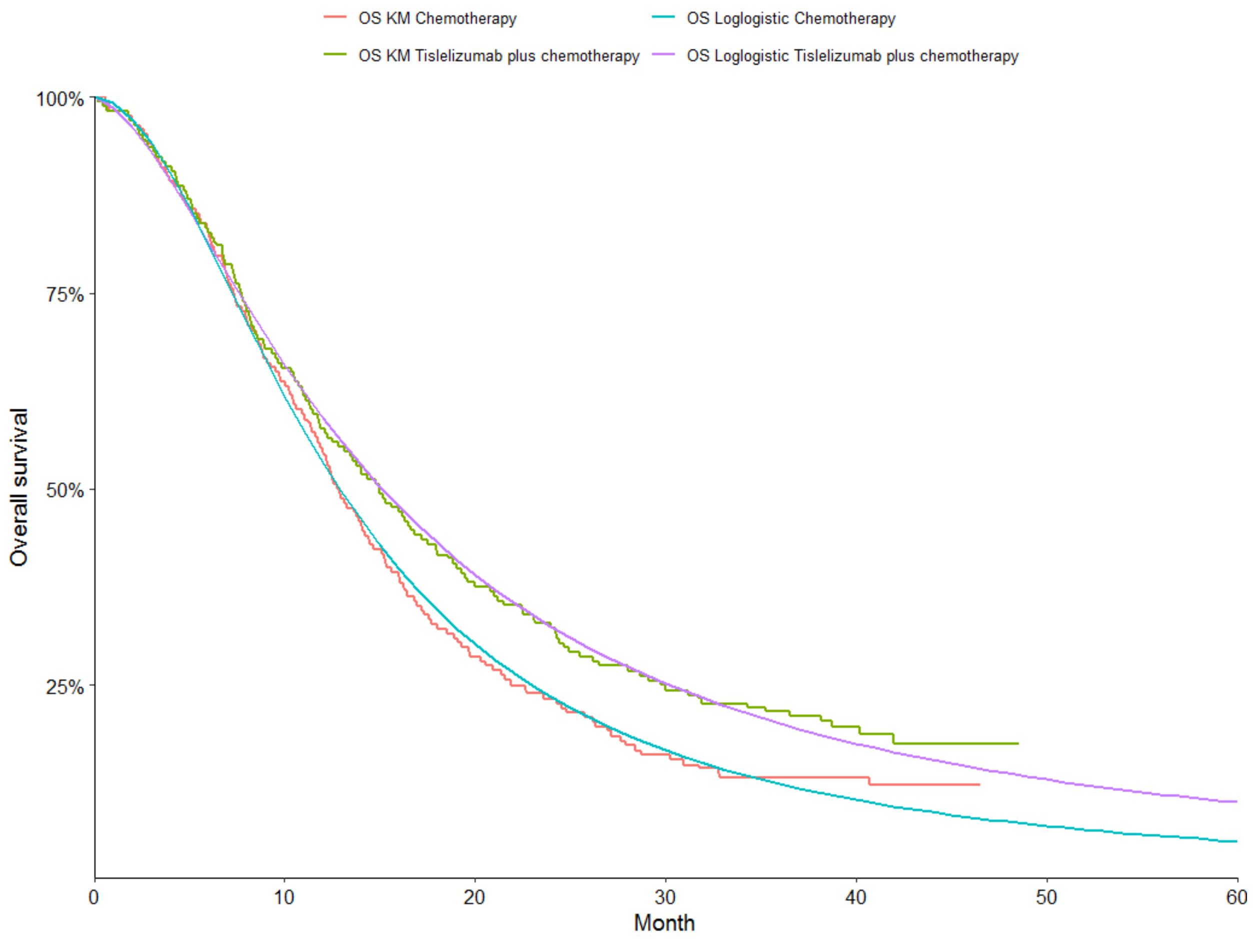

Supplemental Figure 1. The Kaplan-Meier overall survival curves

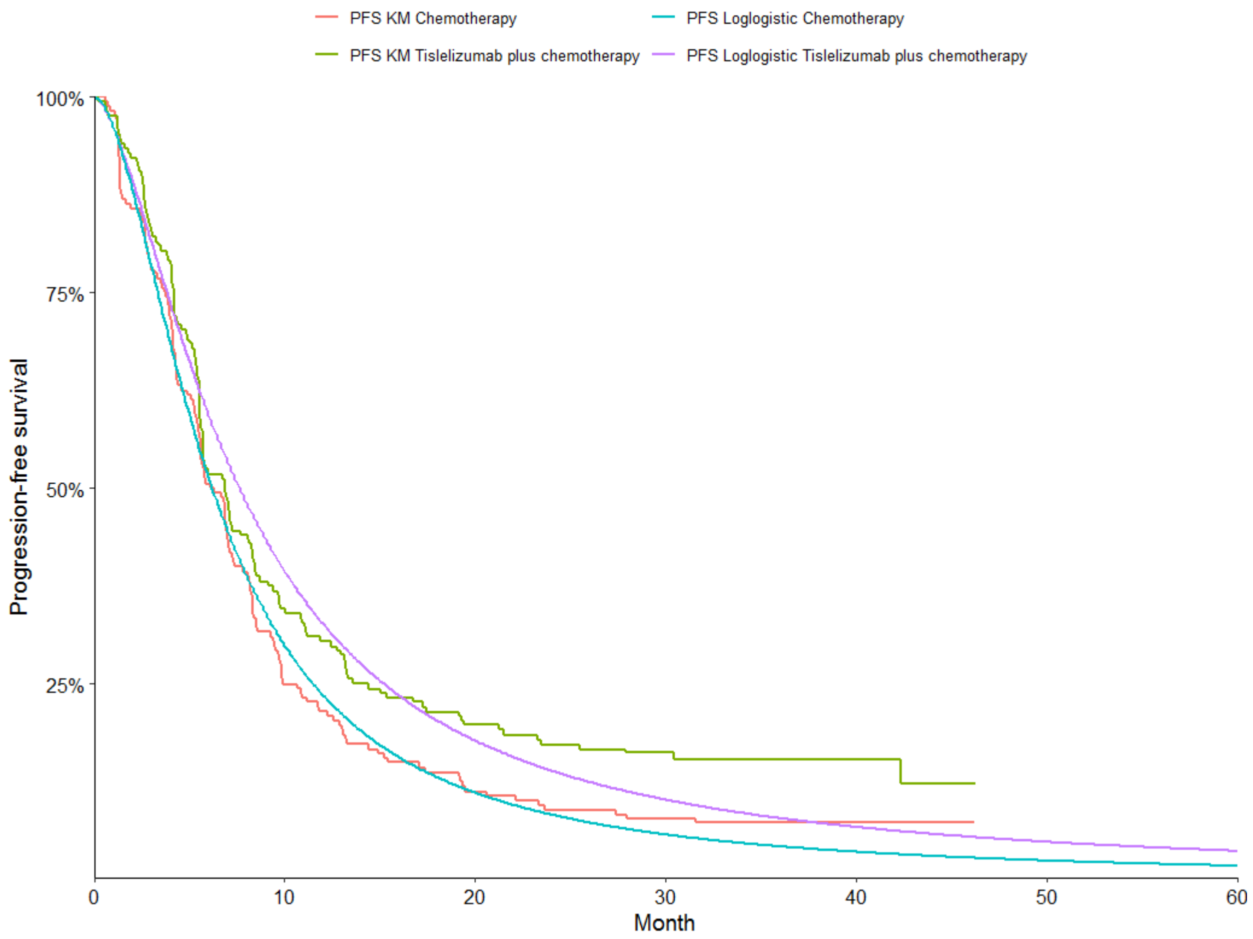

Supplemental Figure 2. The Kaplan-Meier progression-free survival curves

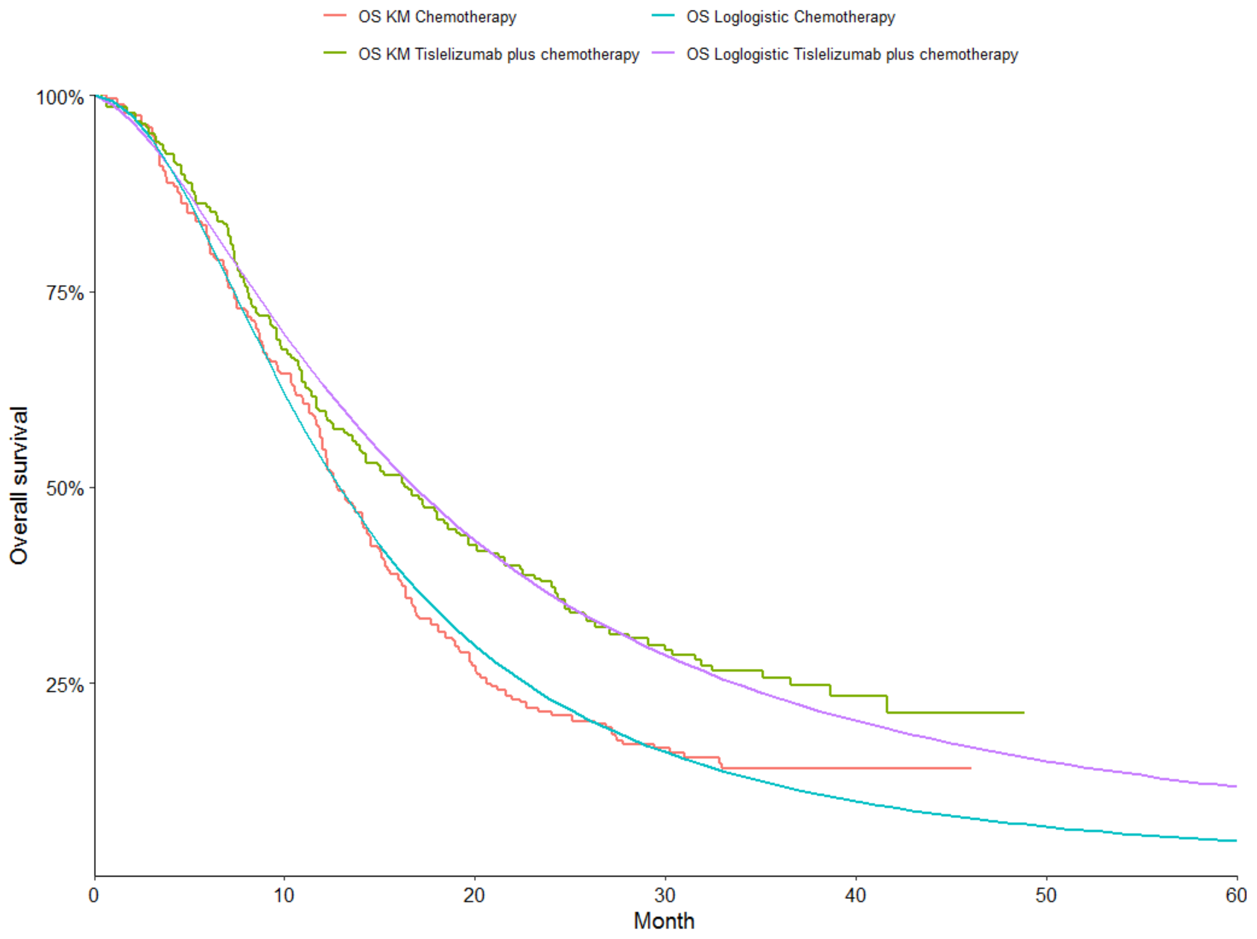

Supplemental Figure 3. The Kaplan-Meier overall survival curves in patients with TAP⩾5%

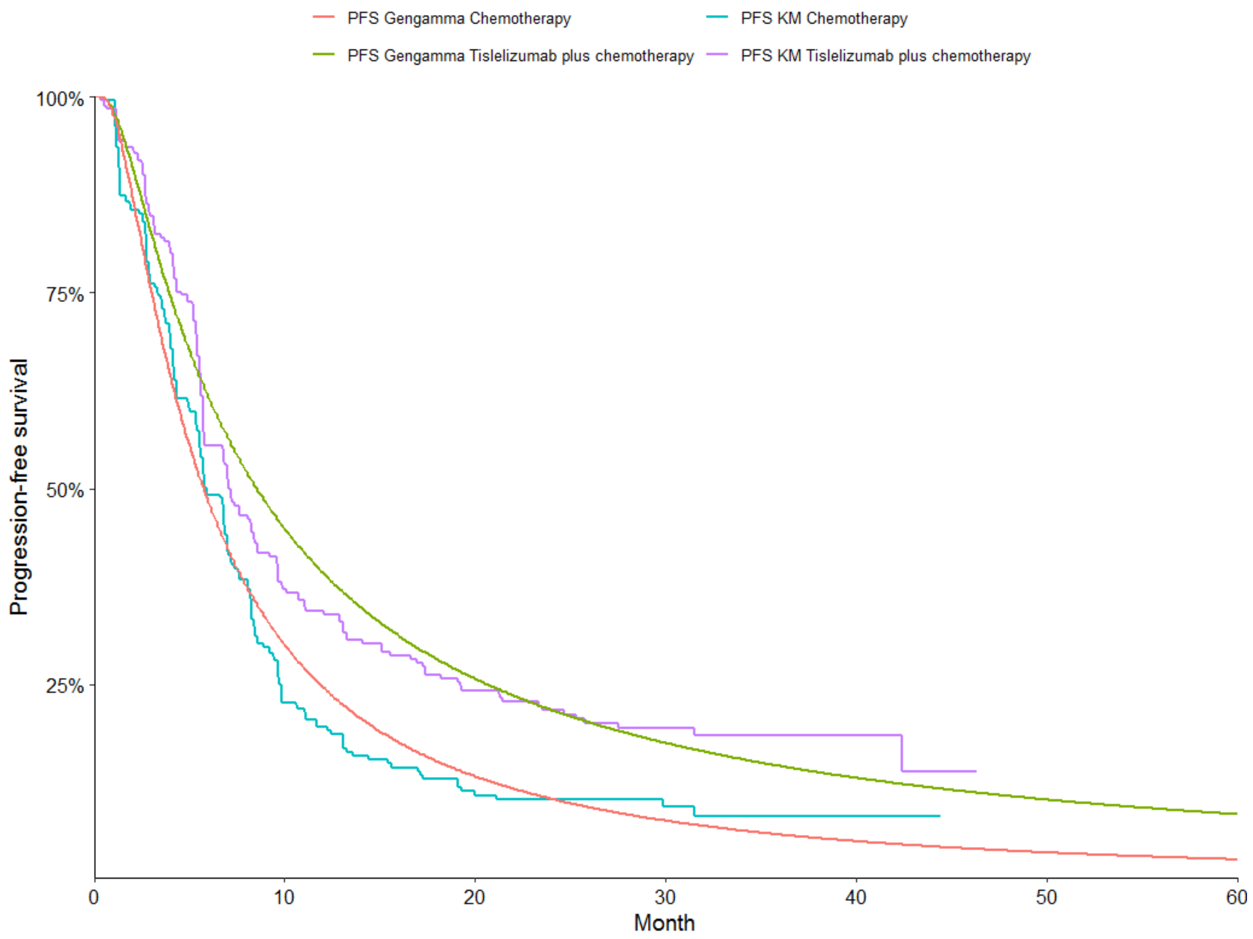

Supplemental Figure 4. The Kaplan-Meier progression-free survival curves in patients with TAP≥5%
